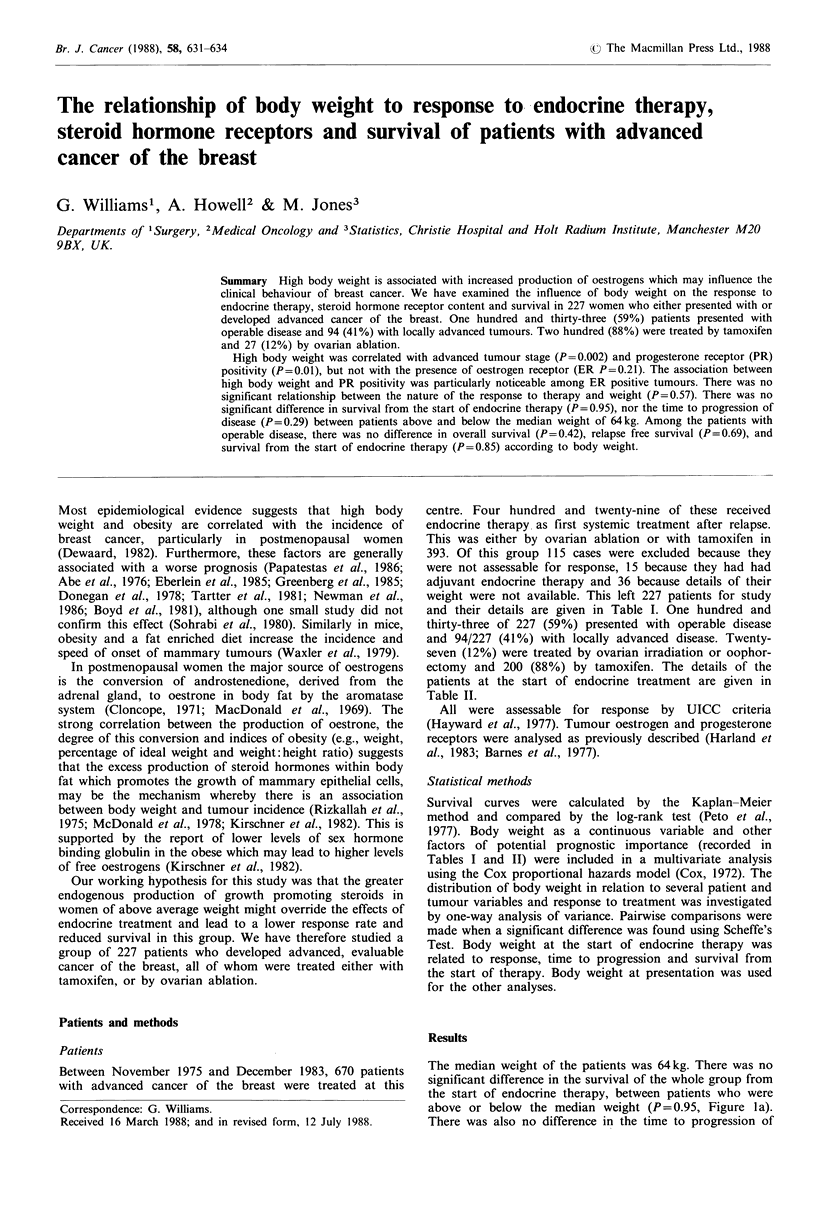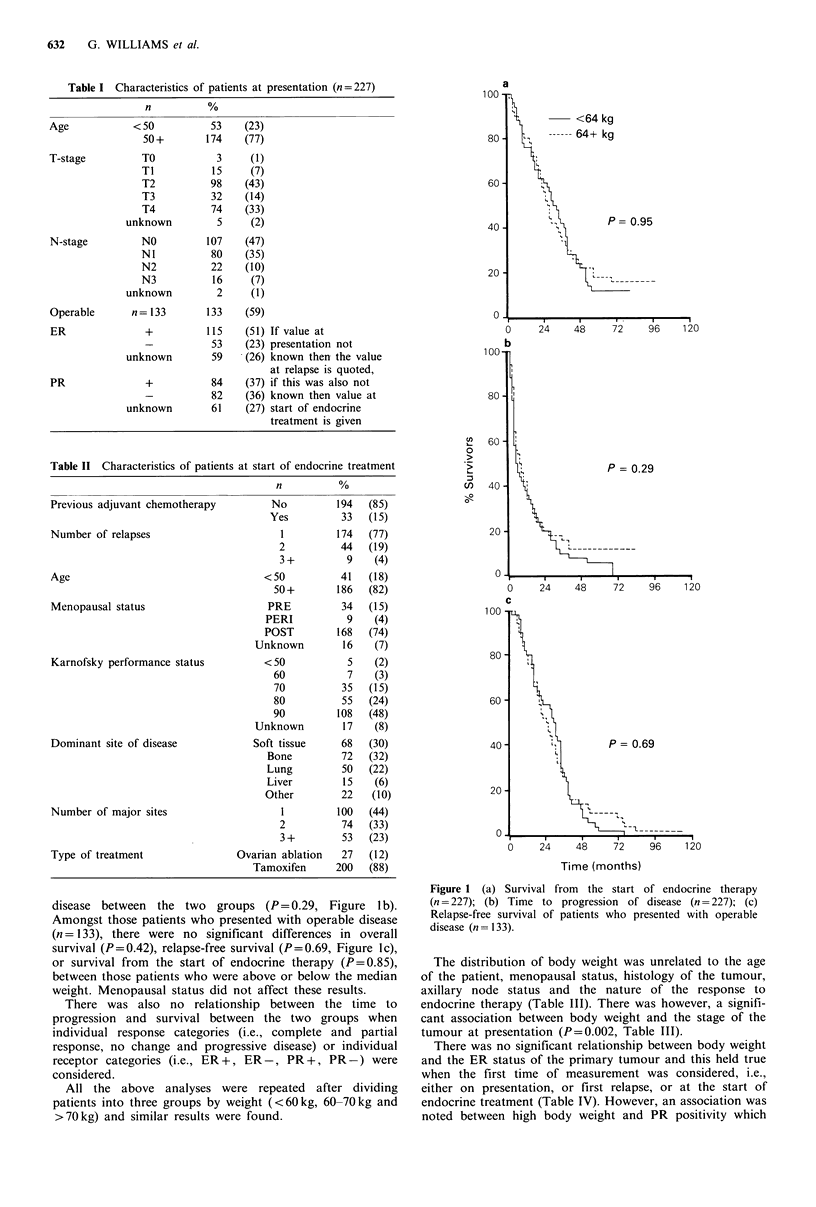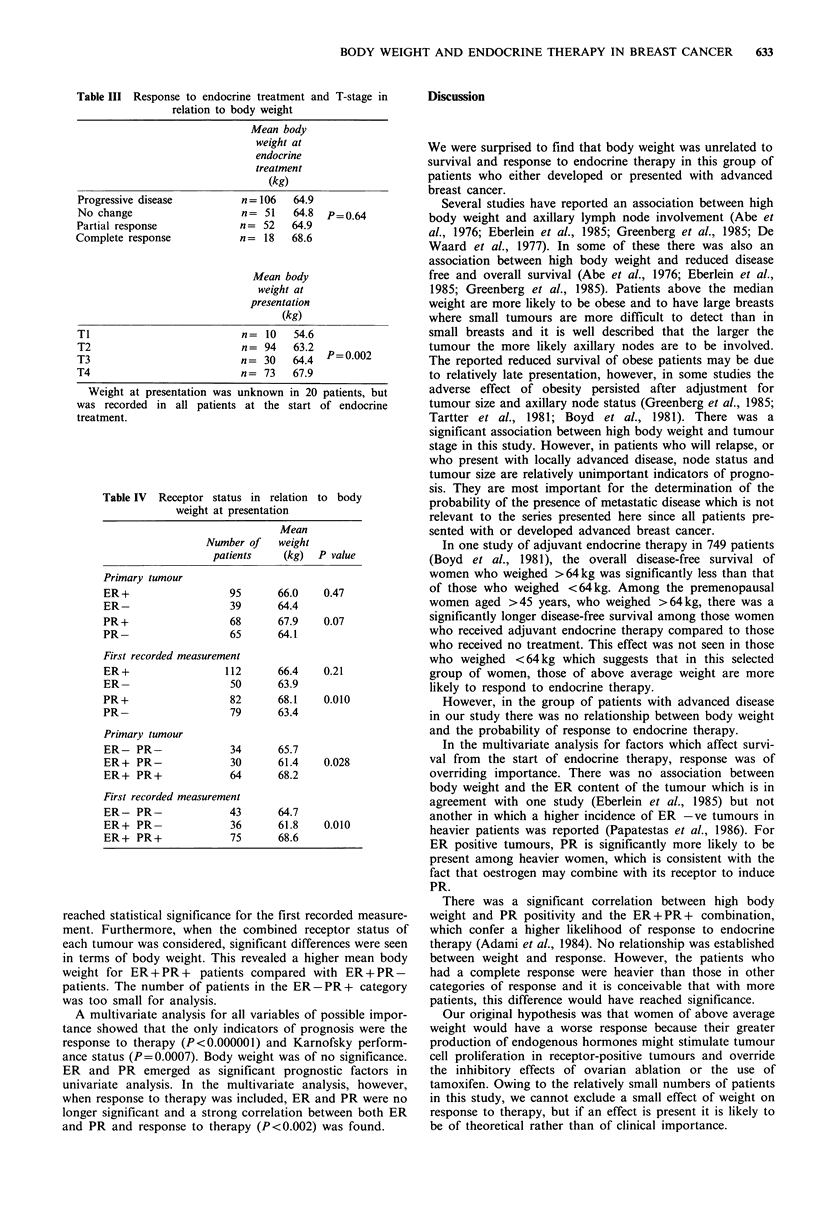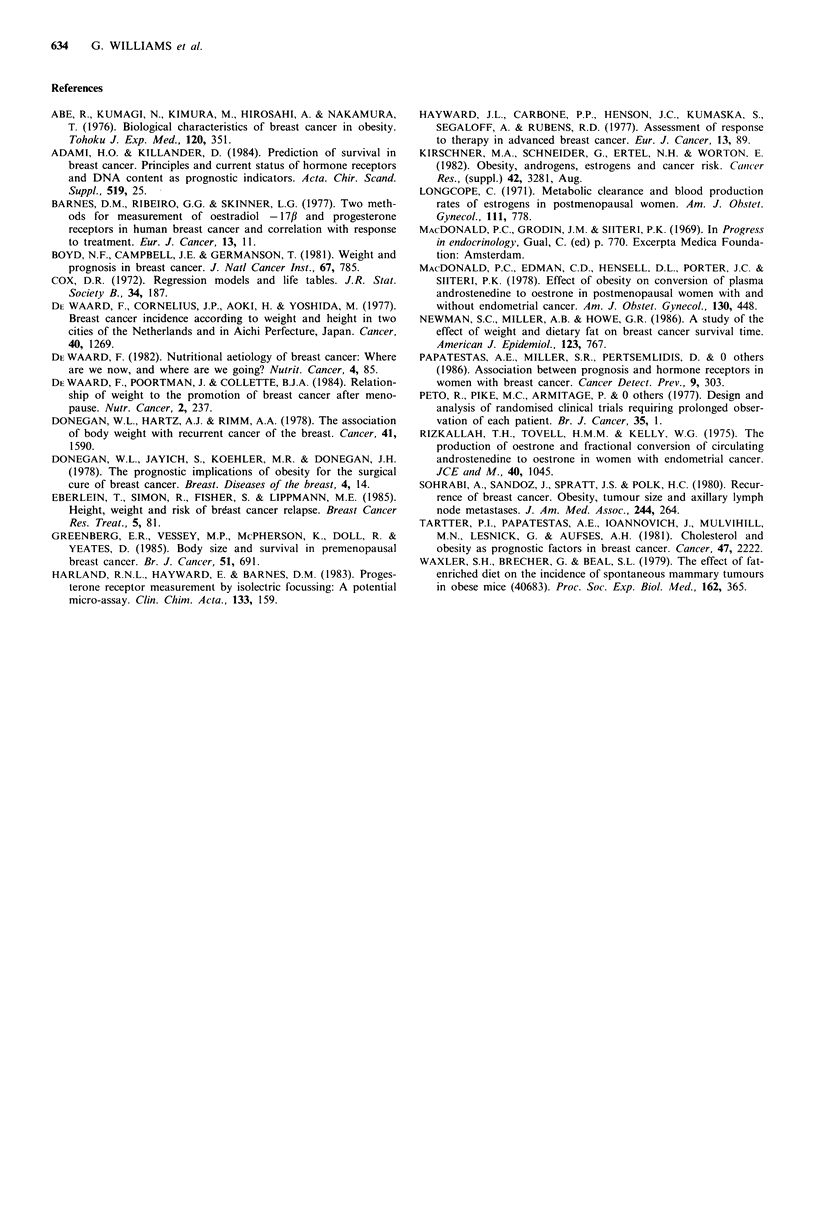# The relationship of body weight to response to endocrine therapy, steroid hormone receptors and survival of patients with advanced cancer of the breast.

**DOI:** 10.1038/bjc.1988.274

**Published:** 1988-11

**Authors:** G. Williams, A. Howell, M. Jones

**Affiliations:** Department of Surgery, Christie Hospital, Manchester, UK.

## Abstract

High body weight is associated with increased production of oestrogens which may influence the clinical behaviour of breast cancer. We have examined the influence of body weight on the response to endocrine therapy, steroid hormone receptor content and survival in 227 women who either presented with or developed advanced cancer of the breast. One hundred and thirty-three (59%) patients presented with operable disease and 94 (41%) with locally advanced tumours. Two hundred (88%) were treated by tamoxifen and 27 (12%) by ovarian ablation. High body weight was correlated with advanced tumour stage (P = 0.002) and progesterone receptor (PR) positivity (P = 0.01), but not with the presence of oestrogen receptor (ER P = 0.21). The association between high body weight and PR positivity was particularly noticeable among ER positive tumours. There was no significant relationship between the nature of the response to therapy and weight (P = 0.57). There was no significant difference in survival from the start of endocrine therapy (P = 0.95), nor the time to progression of disease (P = 0.29) between patients above and below the median weight of 64 kg. Among the patients with operable disease, there was no difference in overall survival (P = 0.42), relapse free survival (P = 0.69), and survival from the start of endocrine therapy (P = 0.85) according to body weight.


*Br. J. Cancer* (1988), **58**, 631–634 © The Macmillan Press Ltd., 1988

### The relationship of body weight to response to endocrine therapy, steroid hormone receptors and survival of patients with advanced cancer of the breast

G. Williams[1], A. Howell[2] & M. Jones[3]

*Departments of [1]Surgery, [2]Medical Oncology and [3]Statistics, Christie Hospital and Holt Radium Institute, Manchester M20 9BX, UK.*

**Summary** High body weight is associated with increased production of oestrogens which may influence the clinical behaviour of breast cancer. We have examined the influence of body weight on the response to endocrine therapy, steroid hormone receptor content and survival in 227 women who either presented with or developed advanced cancer of the breast. One hundred and thirty-three (59%) patients presented with operable disease and 94 (41%) with locally advanced tumours. Two hundred (88%) were treated by tamoxifen and 27 (12%) by ovarian ablation.

High body weight was correlated with advanced tumour stage ($P=0.002$) and progesterone receptor (PR) positivity ($P=0.01$), but not with the presence of oestrogen receptor (ER $P=0.21$). The association between high body weight and PR positivity was particularly noticeable among ER positive tumours. There was no significant relationship between the nature of the response to therapy and weight ($P=0.57$). There was no significant difference in survival from the start of endocrine therapy ($P=0.95$), nor the time to progression of disease ($P=0.29$) between patients above and below the median weight of 64 kg. Among the patients with operable disease, there was no difference in overall survival ($P=0.42$), relapse free survival ($P=0.69$), and survival from the start of endocrine therapy ($P=0.85$) according to body weight.

Most epidemiological evidence suggests that high body weight and obesity are correlated with the incidence of breast cancer, particularly in postmenopausal women (Dewaard, 1982). Furthermore, these factors are generally associated with a worse prognosis (Papatestas *et al.*, 1986; Abe *et al.*, 1976; Eberlein *et al.*, 1985; Greenberg *et al.*, 1985; Donegan *et al.*, 1978; Tartter *et al.*, 1981; Newman *et al.*, 1986; Boyd *et al.*, 1981), although one small study did not confirm this effect (Sohrabi *et al.*, 1980). Similarly in mice, obesity and a fat enriched diet increase the incidence and speed of onset of mammary tumours (Waxler *et al.*, 1979).

In postmenopausal women the major source of oestrogens is the conversion of androstenedione, derived from the adrenal gland, to oestrone in body fat by the aromatase system (Cloncope, 1971; MacDonald *et al.*, 1969). The strong correlation between the production of oestrone, the degree of this conversion and indices of obesity (e.g., weight, percentage of ideal weight and weight:height ratio) suggests that the excess production of steroid hormones within body fat which promotes the growth of mammary epithelial cells, may be the mechanism whereby there is an association between body weight and tumour incidence (Rizkallah *et al.*, 1975; McDonald *et al.*, 1978; Kirschner *et al.*, 1982). This is supported by the report of lower levels of sex hormone binding globulin in the obese which may lead to higher levels of free oestrogens (Kirschner *et al.*, 1982).

Our working hypothesis for this study was that the greater endogenous production of growth promoting steroids in women of above average weight might override the effects of endocrine treatment and lead to a lower response rate and reduced survival in this group. We have therefore studied a group of 227 patients who developed advanced, evaluable cancer of the breast, all of whom were treated either with tamoxifen, or by ovarian ablation.

### Patients and methods

### *Patients*

Between November 1975 and December 1983, 670 patients with advanced cancer of the breast were treated at this centre. Four hundred and twenty-nine of these received endocrine therapy as first systemic treatment after relapse. This was either by ovarian ablation or with tamoxifen in 393. Of this group 115 cases were excluded because they were not assessable for response, 15 because they had had adjuvant endocrine therapy and 36 because details of their weight were not available. This left 227 patients for study and their details are given in Table I. One hundred and thirty-three of 227 (59%) presented with operable disease and 94/227 (41%) with locally advanced disease. Twenty-seven (12%) were treated by ovarian irradiation or oophorectomy and 200 (88%) by tamoxifen. The details of the patients at the start of endocrine treatment are given in Table II.

All were assessable for response by UICC criteria (Hayward *et al.*, 1977). Tumour oestrogen and progesterone receptors were analysed as previously described (Harland *et al.*, 1983; Barnes *et al.*, 1977).

### *Statistical methods*

Survival curves were calculated by the Kaplan–Meier method and compared by the log-rank test (Peto *et al.*, 1977). Body weight as a continuous variable and other factors of potential prognostic importance (recorded in Tables I and II) were included in a multivariate analysis using the Cox proportional hazards model (Cox, 1972). The distribution of body weight in relation to several patient and tumour variables and response to treatment was investigated by one-way analysis of variance. Pairwise comparisons were made when a significant difference was found using Scheffe's Test. Body weight at the start of endocrine therapy was related to response, time to progression and survival from the start of therapy. Body weight at presentation was used for the other analyses.

### Results

The median weight of the patients was 64 kg. There was no significant difference in the survival of the whole group from the start of endocrine therapy, between patients who were above or below the median weight ($P=0.95$, Figure 1a). There was also no difference in the time to progression of

Correspondence: G. Williams.
Received 16 March 1988; and in revised form, 12 July 1988.

**Table I** Characteristics of patients at presentation ($n=227$)

| | n | % | | |
|---|---|---|---|---|
| Age | <50 | 53 | (23) | |
| | 50+ | 174 | (77) | |
| T-stage | T0 | 3 | (1) | |
| | T1 | 15 | (7) | |
| | T2 | 98 | (43) | |
| | T3 | 32 | (14) | |
| | T4 | 74 | (33) | |
| | unknown | 5 | (2) | |
| N-stage | N0 | 107 | (47) | |
| | N1 | 80 | (35) | |
| | N2 | 22 | (10) | |
| | N3 | 16 | (7) | |
| | unknown | 2 | (1) | |
| Operable | $n=133$ | 133 | (59) | |
| ER | + | 115 | (51) | If value at |
| | – | 53 | (23) | presentation not |
| | unknown | 59 | (26) | known then the value at relapse is quoted, |
| PR | + | 84 | (37) | if this was also not |
| | – | 82 | (36) | known then value at |
| | unknown | 61 | (27) | start of endocrine treatment is given |

**Table II** Characteristics of patients at start of endocrine treatment

| | n | % | |
|---|---|---|---|
| Previous adjuvant chemotherapy | No | 194 | (85) |
| | Yes | 33 | (15) |
| Number of relapses | 1 | 174 | (77) |
| | 2 | 44 | (19) |
| | 3+ | 9 | (4) |
| Age | <50 | 41 | (18) |
| | 50+ | 186 | (82) |
| Menopausal status | PRE | 34 | (15) |
| | PERI | 9 | (4) |
| | POST | 168 | (74) |
| | Unknown | 16 | (7) |
| Karnofsky performance status | <50 | 5 | (2) |
| | 60 | 7 | (3) |
| | 70 | 35 | (15) |
| | 80 | 55 | (24) |
| | 90 | 108 | (48) |
| | Unknown | 17 | (8) |
| Dominant site of disease | Soft tissue | 68 | (30) |
| | Bone | 72 | (32) |
| | Lung | 50 | (22) |
| | Liver | 15 | (6) |
| | Other | 22 | (10) |
| Number of major sites | 1 | 100 | (44) |
| | 2 | 74 | (33) |
| | 3+ | 53 | (23) |
| Type of treatment | Ovarian ablation | 27 | (12) |
| | Tamoxifen | 200 | (88) |

**Figure 1** (a) Survival from the start of endocrine therapy ($n=227$); (b) Time to progression of disease ($n=227$); (c) Relapse-free survival of patients who presented with operable disease ($n=133$).

disease between the two groups ($P=0.29$, Figure 1b). Amongst those patients who presented with operable disease ($n=133$), there were no significant differences in overall survival ($P=0.42$), relapse-free survival ($P=0.69$, Figure 1c), or survival from the start of endocrine therapy ($P=0.85$), between those patients who were above or below the median weight. Menopausal status did not affect these results.

There was also no relationship between the time to progression and survival between the two groups when individual response categories (i.e., complete and partial response, no change and progressive disease) or individual receptor categories (i.e., ER+, ER−, PR+, PR−) were considered.

All the above analyses were repeated after dividing patients into three groups by weight (<60 kg, 60–70 kg and >70 kg) and similar results were found.

The distribution of body weight was unrelated to the age of the patient, menopausal status, histology of the tumour, axillary node status and the nature of the response to endocrine therapy (Table III). There was however, a significant association between body weight and the stage of the tumour at presentation ($P=0.002$, Table III).

There was no significant relationship between body weight and the ER status of the primary tumour and this held true when the first time of measurement was considered, i.e., either on presentation, or first relapse, or at the start of endocrine treatment (Table IV). However, an association was noted between high body weight and PR positivity which

**Table III** Response to endocrine treatment and T-stage in relation to body weight

| | | *Mean body weight at endocrine treatment (kg)* | |
|---|---|---|---|
| Progressive disease | $n=106$ | 64.9 | |
| No change | $n=51$ | 64.8 | $P=0.64$ |
| Partial response | $n=52$ | 64.9 | |
| Complete response | $n=18$ | 68.6 | |
| | | *Mean body weight at presentation (kg)* | |
| T1 | $n=10$ | 54.6 | |
| T2 | $n=94$ | 63.2 | |
| T3 | $n=30$ | 64.4 | $P=0.002$ |
| T4 | $n=73$ | 67.9 | |

Weight at presentation was unknown in 20 patients, but was recorded in all patients at the start of endocrine treatment.

**Table IV** Receptor status in relation to body weight at presentation

| | *Number of patients* | *Mean weight (kg)* | *P value* |
|---|---|---|---|
| *Primary tumour* | | | |
| ER+ | 95 | 66.0 | 0.47 |
| ER− | 39 | 64.4 | |
| PR+ | 68 | 67.9 | 0.07 |
| PR− | 65 | 64.1 | |
| *First recorded measurement* | | | |
| ER+ | 112 | 66.4 | 0.21 |
| ER− | 50 | 63.9 | |
| PR+ | 82 | 68.1 | 0.010 |
| PR− | 79 | 63.4 | |
| *Primary tumour* | | | |
| ER− PR− | 34 | 65.7 | |
| ER+ PR− | 30 | 61.4 | 0.028 |
| ER+ PR+ | 64 | 68.2 | |
| *First recorded measurement* | | | |
| ER− PR− | 43 | 64.7 | |
| ER+ PR− | 36 | 61.8 | 0.010 |
| ER+ PR+ | 75 | 68.6 | |

reached statistical significance for the first recorded measurement. Furthermore, when the combined receptor status of each tumour was considered, significant differences were seen in terms of body weight. This revealed a higher mean body weight for ER+PR+ patients compared with ER+PR− patients. The number of patients in the ER−PR+ category was too small for analysis.

A multivariate analysis for all variables of possible importance showed that the only indicators of prognosis were the response to therapy ($P<0.000001$) and Karnofsky performance status ($P=0.0007$). Body weight was of no significance. ER and PR emerged as significant prognostic factors in univariate analysis. In the multivariate analysis, however, when response to therapy was included, ER and PR were no longer significant and a strong correlation between both ER and PR and response to therapy ($P<0.002$) was found.

### Discussion

We were surprised to find that body weight was unrelated to survival and response to endocrine therapy in this group of patients who either developed or presented with advanced breast cancer.

Several studies have reported an association between high body weight and axillary lymph node involvement (Abe *et al.*, 1976; Eberlein *et al.*, 1985; Greenberg *et al.*, 1985; De Waard *et al.*, 1977). In some of these there was also an association between high body weight and reduced disease free and overall survival (Abe *et al.*, 1976; Eberlein *et al.*, 1985; Greenberg *et al.*, 1985). Patients above the median weight are more likely to be obese and to have large breasts where small tumours are more difficult to detect than in small breasts and it is well described that the larger the tumour the more likely axillary nodes are to be involved. The reported reduced survival of obese patients may be due to relatively late presentation, however, in some studies the adverse effect of obesity persisted after adjustment for tumour size and axillary node status (Greenberg *et al.*, 1985; Tartter *et al.*, 1981; Boyd *et al.*, 1981). There was a significant association between high body weight and tumour stage in this study. However, in patients who will relapse, or who present with locally advanced disease, node status and tumour size are relatively unimportant indicators of prognosis. They are most important for the determination of the probability of the presence of metastatic disease which is not relevant to the series presented here since all patients presented with or developed advanced breast cancer.

In one study of adjuvant endocrine therapy in 749 patients (Boyd *et al.*, 1981), the overall disease-free survival of women who weighed >64 kg was significantly less than that of those who weighed <64 kg. Among the premenopausal women aged >45 years, who weighed >64 kg, there was a significantly longer disease-free survival among those women who received adjuvant endocrine therapy compared to those who received no treatment. This effect was not seen in those who weighed <64 kg which suggests that in this selected group of women, those of above average weight are more likely to respond to endocrine therapy.

However, in the group of patients with advanced disease in our study there was no relationship between body weight and the probability of response to endocrine therapy.

In the multivariate analysis for factors which affect survival from the start of endocrine therapy, response was of overriding importance. There was no association between body weight and the ER content of the tumour which is in agreement with one study (Eberlein *et al.*, 1985) but not another in which a higher incidence of ER −ve tumours in heavier patients was reported (Papatestas *et al.*, 1986). For ER positive tumours, PR is significantly more likely to be present among heavier women, which is consistent with the fact that oestrogen may combine with its receptor to induce PR.

There was a significant correlation between high body weight and PR positivity and the ER+PR+ combination, which confer a higher likelihood of response to endocrine therapy (Adami *et al.*, 1984). No relationship was established between weight and response. However, the patients who had a complete response were heavier than those in other categories of response and it is conceivable that with more patients, this difference would have reached significance.

Our original hypothesis was that women of above average weight would have a worse response because their greater production of endogenous hormones might stimulate tumour cell proliferation in receptor-positive tumours and override the inhibitory effects of ovarian ablation or the use of tamoxifen. Owing to the relatively small numbers of patients in this study, we cannot exclude a small effect of weight on response to therapy, but if an effect is present it is likely to be of theoretical rather than of clinical importance.

### References

ABE, R., KUMAGI, N., KIMURA, M., HIROSAHI, A. & NAKAMURA, T. (1976). Biological characteristics of breast cancer in obesity. *Tohoku J. Exp. Med.*, **120**, 351.

ADAMI, H.O. & KILLANDER, D. (1984). Prediction of survival in breast cancer. Principles and current status of hormone receptors and DNA content as prognostic indicators. *Acta. Chir. Scand. Suppl.*, **519**, 25.

BARNES, D.M., RIBEIRO, G.G. & SKINNER, L.G. (1977). Two methods for measurement of oestradiol $-17\beta$ and progesterone receptors in human breast cancer and correlation with response to treatment. *Eur. J. Cancer*, **13**, 11.

BOYD, N.F., CAMPBELL, J.E. & GERMANSON, T. (1981). Weight and prognosis in breast cancer. *J. Natl Cancer Inst.*, **67**, 785.

COX, D.R. (1972). Regression models and life tables. *J.R. Stat. Society B.*, **34**, 187.

DE WAARD, F., CORNELIUS, J.P., AOKI, H. & YOSHIDA, M. (1977). Breast cancer incidence according to weight and height in two cities of the Netherlands and in Aichi Perfecture, Japan. *Cancer*, **40**, 1269.

DE WAARD, F. (1982). Nutritional aetiology of breast cancer: Where are we now, and where are we going? *Nutrit. Cancer*, **4**, 85.

DE WAARD, F., POORTMAN, J. & COLLETTE, B.J.A. (1984). Relationship of weight to the promotion of breast cancer after menopause. *Nutr. Cancer*, **2**, 237.

DONEGAN, W.L., HARTZ, A.J. & RIMM, A.A. (1978). The association of body weight with recurrent cancer of the breast. *Cancer*, **41**, 1590.

DONEGAN, W.L., JAYICH, S., KOEHLER, M.R. & DONEGAN, J.H. (1978). The prognostic implications of obesity for the surgical cure of breast cancer. *Breast. Diseases of the breast*, **4**, 14.

EBERLEIN, T., SIMON, R., FISHER, S. & LIPPMANN, M.E. (1985). Height, weight and risk of breast cancer relapse. *Breast Cancer Res. Treat.*, **5**, 81.

GREENBERG, E.R., VESSEY, M.P., MCPHERSON, K., DOLL, R. & YEATES, D. (1985). Body size and survival in premenopausal breast cancer. *Br. J. Cancer*, **51**, 691.

HARLAND, R.N.L., HAYWARD, E. & BARNES, D.M. (1983). Progesterone receptor measurement by isolectric focussing: A potential micro-assay. *Clin. Chim. Acta.*, **133**, 159.

HAYWARD, J.L., CARBONE, P.P., HENSON, J.C., KUMASKA, S., SEGALOFF, A. & RUBENS, R.D. (1977). Assessment of response to therapy in advanced breast cancer. *Eur. J. Cancer*, **13**, 89.

KIRSCHNER, M.A., SCHNEIDER, G., ERTEL, N.H. & WORTON, E. (1982). Obesity, androgens, estrogens and cancer risk. *Cancer Res.*, (suppl.) **42**, 3281, Aug.

LONGCOPE, C. (1971). Metabolic clearance and blood production rates of estrogens in postmenopausal women. *Am. J. Obstet. Gynecol.*, **111**, 778.

MACDONALD, P.C., GRODIN, J.M. & SIITERI, P.K. (1969). In *Progress in endocrinology*, Gual, C. (ed) p. 770. Excerpta Medica Foundation: Amsterdam.

MACDONALD, P.C., EDMAN, C.D., HENSELL, D.L., PORTER, J.C. & SIITERI, P.K. (1978). Effect of obesity on conversion of plasma androstenedine to oestrone in postmenopausal women with and without endometrial cancer. *Am. J. Obstet. Gynecol.*, **130**, 448.

NEWMAN, S.C., MILLER, A.B. & HOWE, G.R. (1986). A study of the effect of weight and dietary fat on breast cancer survival time. *American J. Epidemiol.*, **123**, 767.

PAPATESTAS, A.E., MILLER, S.R., PERTSEMLIDIS, D. & 0 others (1986). Association between prognosis and hormone receptors in women with breast cancer. *Cancer Detect. Prev.*, **9**, 303.

PETO, R., PIKE, M.C., ARMITAGE, P. & 0 others (1977). Design and analysis of randomised clinical trials requiring prolonged observation of each patient. *Br. J. Cancer*, **35**, 1.

RIZKALLAH, T.H., TOVELL, H.M.M. & KELLY, W.G. (1975). The production of oestrone and fractional conversion of circulating androstenedine to oestrone in women with endometrial cancer. *JCE and M.*, **40**, 1045.

SOHRABI, A., SANDOZ, J., SPRATT, J.S. & POLK, H.C. (1980). Recurrence of breast cancer. Obesity, tumour size and axillary lymph node metastases. *J. Am. Med. Assoc.*, **244**, 264.

TARTTER, P.I., PAPATESTAS, A.E., IOANNOVICH, J., MULVIHILL, M.N., LESNICK, G. & AUFSES, A.H. (1981). Cholesterol and obesity as prognostic factors in breast cancer. *Cancer*, **47**, 2222.

WAXLER, S.H., BRECHER, G. & BEAL, S.L. (1979). The effect of fat-enriched diet on the incidence of spontaneous mammary tumours in obese mice (40683). *Proc. Soc. Exp. Biol. Med.*, **162**, 365.